# Targeting the AGEs-RAGE axis: pathogenic mechanisms and therapeutic interventions in diabetic wound healing

**DOI:** 10.3389/fmed.2025.1667620

**Published:** 2025-09-18

**Authors:** Haohui Lin, Yi Yang, Xia Wang, Manhon Chung, Li Zhang, Sa Cai, Xiaohua Pan, Yu Pan

**Affiliations:** ^1^Laboratory of Translational and Regenerative Medicine, The 2nd Affiliated Hospital of Shenzhen University, Medical School, Shenzhen University, Shenzhen, China; ^2^School of Medicine, The Chinese University of Hong Kong Shenzhen, Shenzhen, China; ^3^Department of Plastic and Reconstructive Surgery, Shanghai Ninth People’s Hospital, Shanghai Jiao Tong University School of Medicine, Shanghai, China

**Keywords:** advanced glycation end products, receptor for advanced glycation end products, inflammation, diabetes, wound healing, diabetic wound

## Abstract

Diabetes is a global health problem, with diabetic wounds constituting one of its most severe complications. Advanced glycation end products (AGEs) and their receptor, the receptor for advanced glycation end products (RAGE), play a key role in the pathogenesis of diabetic wounds. Accumulated AGEs bind to RAGE, activating various inflammatory and oxidative stress pathways such as NF-κB, PI3K-AKT, and JAK–STAT signaling, impairing normal wound healing. This review describes mechanisms by which the AGEs-RAGE axis disrupts vascular function, immune regulation, and cellular regeneration, thereby driving the formation of chronic non-healing wounds. Furthermore, we discuss emerging therapeutic strategies targeting the AGEs-RAGE axis, such as selective RAGE inhibitors, monoclonal antibodies, gene-based interventions, and AGE scavengers, highlighting their potential to enhance the treatment of diabetic chronic wounds.

## 1 Introduction

The escalating diabetic patients imposes a substantial socioeconomic burden worldwide. Global prevalence of diabetes was estimated at 10.5% (536.6 million) in 2021, with a projected rise to 12.2% (783.2 million) by 2045 (1). Diabetes-related complications represent the primary cause of disability and mortality among diabetic patients. Among these chronic complications, diabetic wounds stand out as one of the most life-threatening conditions, demonstrating strong associations with hospitalization, limb amputation, and increased mortality rates (2). Epidemiological data reveal that approximately 18.6 million people with diabetes develop a foot ulcer (the most common type of diabetic wound) each year (3). Currently, no definitive therapeutic approach exists for the effective repair of diabetic wounds. A comprehensive understanding of the pathogenic mechanisms underlying diabetic wound formation may reveal novel therapeutic targets for this debilitating condition.

Wound healing is a highly orchestrated and complex process. Normal wounds (or acute wounds) healing typically contains four consecutive and overlapping stages: (a) Hemostasis stage: Vascular constriction occurs, with rapid recruitment of platelets and clotting factors to seal the wound; (b) Inflammation stage: Innate immune cells, such as neutrophils and macrophages, migrate to the wound in response to inflammatory cytokines, pathogen-associated molecular patterns (PAMPs) or danger-associated molecular patterns (DAMPs), and eliminate pathogens and necrotic cells; (c) Proliferation stage: As inflammation resolves, endothelial cells, fibroblasts, and keratinocytes proliferate and migrate from the wound margins, forming granulation tissue and regenerating the epidermis for wound closure; (d) Remodeling phase: The structure and composition of granulation tissue was remodeled. Some newly generated vessels atrophy, and collagen continues to deposit, ultimately leading to the formation of scars (4).

In diabetic wounds, pathological factors such as bacterial infections, oxidative stress, local ischemia, persistent inflammation, and skin nerve atrophy collectively disrupt the normal healing cascade, resulting in chronic non-healing wounds. Signaling pathways mediating inflammation and oxidative stress are closely associated with these pathological alterations (5). Advanced glycation end products (AGEs) are a class of heterogeneous glycated derivatives that exert biological effects primarily through binding to the Receptor for Advanced Glycation End Products (RAGE). This interaction activates various downstream pro-inflammatory and oxidative stress pathways, including the Nuclear Factor Kappa B (NF-κB), Phosphoinositide 3-kinase/Protein kinase B (PI3K/AKT), and Janus Kinase/Signal Transducer and Activator of Transcription (JAK/STAT) signaling pathways. Numerous chronic, inflammatory diseases, such as neurodegenerative disorders, diabetes, atherosclerosis, and cancers, are associated with the persistent activity of the AGEs-RAGE axis, indicating its potential critical role in the pathogenesis of diabetic wounds (6, 7).

In this review, we introduce the structure and function of AGEs and their receptor RAGE. Furthermore, we emphasize the contribution of the AGEs-RAGE axis in diabetic wounds and discuss therapeutic strategies targeting this pathway, aiming to provide innovative perspectives for enhancing diabetic wound healing.

## 2 AGEs and RAGE

### 2.1 The formation and accumulation of AGEs

AGEs represent a highly heterogeneous class of compounds produced through a series of non-enzymatic reactions, known as the Maillard reaction (8). This reaction consists of three major steps: glycation, Amadori rearrangement, and non-enzymatic peptide cross-linking. Initially, reducing sugars react reversibly with the amino groups of proteins, lipids, and nucleic acids, forming early Schiff bases. In high-glucose systems, the rate of this reaction is significantly enhanced. Subsequently, the Schiff bases undergo slow, irreversible rearrangement to form Amadori products. Finally, these Amadori products undergo a series of complex reactions, including oxidation, dehydration, and cross-linking, which yield various types of AGEs (9, 10).

AGEs accumulate in the body *via* both exogenous intake and endogenous synthesis. Exogenous intake is the primary contributor to the total body burden of AGEs, predominantly derived from high-temperature-cooked foods, sugary beverages, and tobacco products (8). Following absorption, externally derived AGEs can bind to various proteins and tissues throughout the body. Endogenous AGEs mainly arise from post-translational glycation of proteins, exhibiting significant cellular toxicities (11). In healthy individuals, accumulated AGEs are efficiently cleared through multiple pathways. The ubiquitin–proteasome system and autophagy are responsible for the quality control of protein, selectively degrading damaged proteins to maintain cellular homeostasis (12). Moreover, low-molecular-weight AGEs can be excreted renally (13). Furthermore, the glyoxalase system helps reduce AGEs formation by catalyzing reducing sugar into non-toxic metabolites (14). However, under conditions such as chronic high-AGE diets or pathological states, such as aging, oxidative stress, hyperglycemia, and hyperlipidemia, the equilibrium between AGE formation and clearance becomes disrupted (15). Over time, increasing AGEs accumulate in tissues. Their presence triggers inflammatory responses and oxidative stress through persistent interaction with RAGE and additionally induces protein dysfunction through cross-linking-mediated structural alterations (15).

### 2.2 Structure and isoforms of RAGE

**Figure 1:**
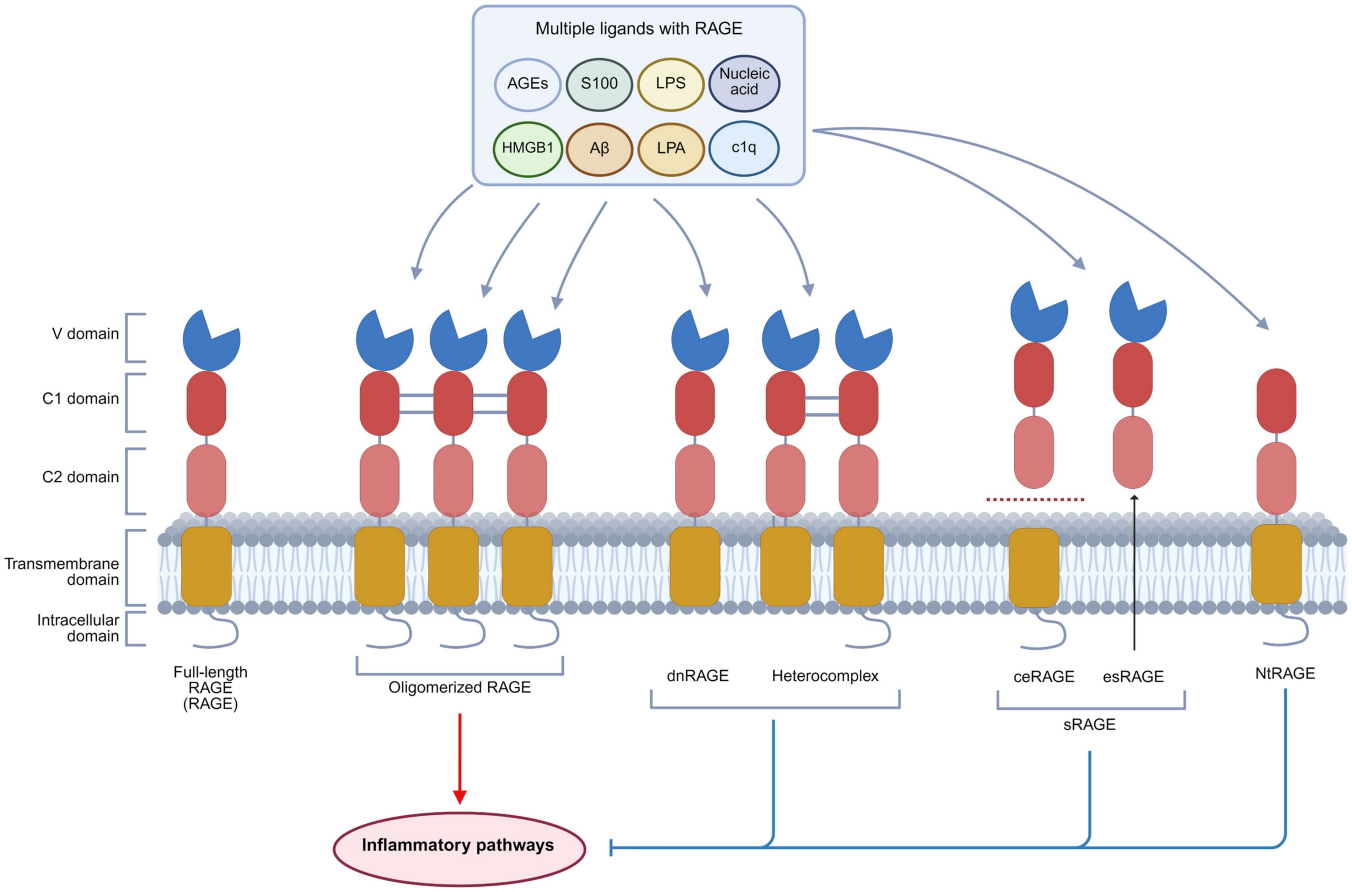
Basic structure and isoforms of RAGE. RAGE exists in four principal isoforms: Full-length RAGE, dnRAGE, sRAGE, and NtRAGE. Full-length RAGE consists of an extracellular domain (V, C1, and C2 domains), a transmembrane domain, and an intracellular domain. It is capable of binding multiple ligands, including AGEs, HMGB1, S100 proteins, Aβ, LPA, LPS, C1q, and nucleic acids, and requires oligomerization for signal transduction. dnRAGE, sRAGE, and NtRAGE lack complete structural domains. Upon ligand binding, they fail to activate downstream inflammatory pathways, thus functioning as signaling inhibitors. This figure was created in https://BioRender.com.

The RAGE gene is located within the histocompatibility complex (MHC) class III region on human chromosome 6 and contains 11 exons and 10 introns. The term “RAGE” generally refers to the full-length RAGE isoform, a transmembrane glycoprotein consisting of 404 amino acids with a molecular weight of approximately 50 kDa. It is recognized as the only isoform capable of transducing signals (16). Structurally, RAGE is divided into three distinct regions: the extracellular, transmembrane segment, and intracellular domain. The extracellular segment encompasses an immunoglobulin-like variable (V) domain and two constant-type domains (C1 and C2). The transmembrane segment includes a helical domain, and the intracellular domain contains a highly charged, disordered cytoplasmic tail. The V and C1 domains function as ligand-binding interfaces, characterized by a highly positively charged region that interacts with negatively charged regions of ligands (17–19). This binding mechanism is a hallmark of pattern recognition receptors (PRRs), enabling RAGE to engage with a broad spectrum of structurally diverse ligands, such as AGEs, high mobility group box 1 (HMGB1), S100 proteins, amyloid beta (Aβ), lipopolysaccharides (LPS), lysophosphatidic acid (LPA), nucleic acids, and complement protein C1q (20) (Figure 1).

RAGE exists in four main isoforms: the transmembrane signaling receptor (full-length RAGE, RAGE), the dominant negative form (dnRAGE), the circulating soluble forms (sRAGE), and the N-truncated form (NtRAGE) (21). RAGE can form oligomers on the plasma membrane, and the preassembly has substantial implications for its activation mechanism. This process is driven by heparan sulfate scaffolds on the cell surface, which facilitate RAGE oligomerization into signaling-competent complexes (22, 23). The dnRAGE isoform, which lacks the cytoplasmic domain, disrupts signal transduction and may form nonfunctional heterocomplexes with full-length RAGE (23–25). sRAGE contains cleaved RAGE (ceRAGE) and endogenous secreted RAGE (esRAGE). Lacking transmembrane and intracellular segments, sRAGE circulates in the serum and competitively binds RAGE ligands to inhibit the activation of RAGE (26). Moreover, sRAGE is considered a biomarker for inflammatory diseases (27). The NtRAGE isoform, which lacks the V domain, functions as a decoy receptor to modulate the RAGE signaling activity (28, 29) (Figure 1).

### 2.3 Biological function and distribution of RAGE

**Figure 2:**
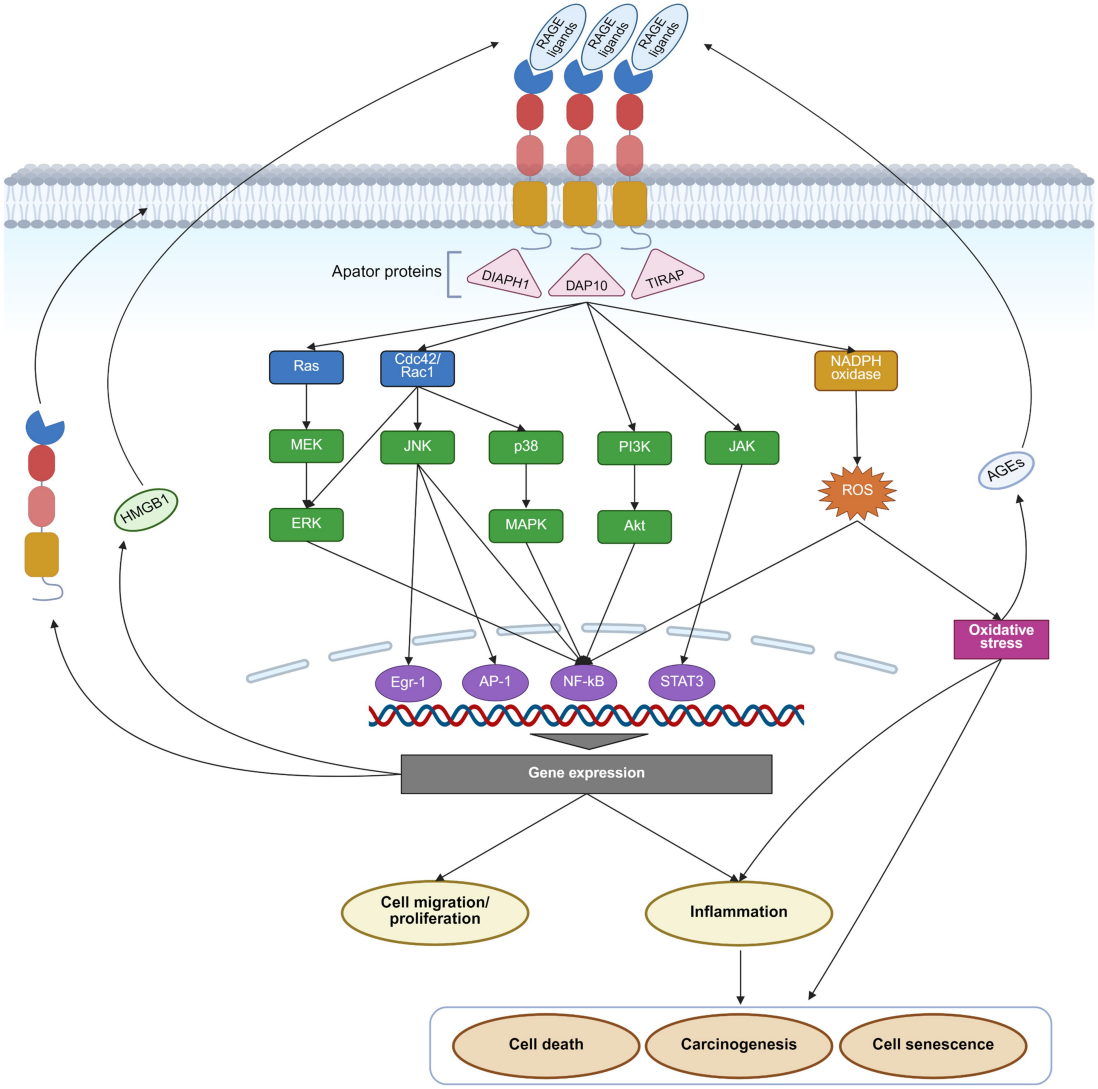
RAGE pathway and its effects. Upon binding its ligands, RAGE activates Ras/MEK/ERK, p38/MAPK, JAK/STAT, PI3K/AKT, and Cdc42/Rac1 signaling pathways, along with NADPH oxidase through adaptor proteins such as DIAPH1, DAP10, and TIRAP. These downstream pathways subsequently activate transcription factors such as NF-κB, AP1, Egr1, and STAT3, driving gene expression and triggering a cascade of effects, including inflammation, cell migration, proliferation, senescence, death, and carcinogenesis. Moreover, activation of the RAGE pathway enhances the expression of RAGE itself and several of its ligands, like AGEs and HMGB1, creating a positive feedback loop that further amplifies RAGE signaling. This figure was created in https://BioRender.com.

The primary biological function of RAGE is to act as a receptor that transduces ligand signals, initiating a cascade of downstream biochemical reactions. The cytoplasmic tail of RAGE lacks intrinsic kinase activity and therefore depends on adaptor proteins, such as the Formin homology 1 domain of diaphanous-1 (DIAPH1), DNAX-activating Protein 10 (DAP10), and Toll-interleukin 1 receptor domain containing adaptor protein (TIRAP) to initiate intracellular signaling (30–32). These adaptors subsequently activate multiple signaling pathways, including Ras/mitogen-activated extracellular signal-regulated kinase (MEK)/extracellular regulated protein kinases (ERK), mitogen-activated protein kinase (MAPK)/P38, JAK/STAT, PI3K/AKT, and cell division cycle 42 (Cdc42)/Rac family small GTPase 1 (Rac1) (20, 33). Downstream transcription factors, including NF-κB, STAT3, activator protein 1 (AP1), and early growth response 1 (Egr1), promote the secretion of inflammatory cytokines like interleukin (IL)-1, IL-6, tumor necrosis factor, interferon, and chemokines, and thereby regulate cell proliferation and migration, and induce apoptosis (33). Moreover, RAGE activation facilitates the production of reactive oxygen species (ROS) by inflammatory signaling pathways and the enhancement of the activity of nicotinamide adenine dinucleotide phosphate hydrogen (NADPH) oxidase (Figure 2).

RAGE is widely expressed across multiple cell types. Under physiological conditions, RAGE is expressed at low levels in several cell types, including monocytes/macrophages, T-lymphocytes, epithelial cells, endothelial cells, dendritic cells, fibroblasts, smooth muscle cells, neuronal cells, glial cells, and chondrocytes (34–37). On the contrary, in pathophysiological settings, such as diabetes, chronic inflammation, cancer, or neurodegenerative disorders, RAGE expression is increased drastically (38). This upregulation is mediated by an NF-κB-dependent positive feedback mechanism. Specifically, ligand-induced RAGE activation potently stimulates NF-κB, which then binds to the RAGE promoter to enhance its transcription, thereby creating a vicious cycle that amplifies RAGE signaling (39). Furthermore, inflammatory response and oxidative stress promote the production of RAGE ligands, including AGEs and HMGB1, further amplifying the effects of the RAGE pathway (40, 41) (Figure 2). Therapeutic strategies aimed at suppressing inflammation and oxidative stress, clearing RAGE ligands, or directly inhibiting RAGE may present viable strategies to disrupt this detrimental positive feedback loop (42–45). For instance, clearance can be achieved by sRAGE or ligand scavengers like aminoguanidine, paquinimod, and glycyrrhizin (42, 44–46).

RAGE acts as a double-edged modulator in maintaining homeostasis. It exerts indispensable physiological roles while also driving pathological damage when dysregulated. During embryonic development, RAGE supports alveolar epithelial cell differentiation and the growth and migration of cerebral neurons (47, 48). As a PRR, it binds microbial ligands, initiates innate immune responses, and promotes immune cells to eliminate pathogens and apoptotic cells (49, 50). Moreover, moderate RAGE activation-induced recruitment of immune cells contributes to the regeneration of skeletal muscles and peripheral nerves (51, 52). However, in pathological conditions, excessive activation of RAGE results in chronic inflammation, excessive oxidative stress, and cellular dysfunction, which collectively contribute to disease progression (53). Aberrant RAGE signaling is implicated in various chronic inflammatory diseases, including diabetes, atherosclerosis, nephropathy, pathological scar, neurodegenerative disorders, and cancer (54–57). Notably, RAGE also demonstrates non-receptor effects. When phosphorylated by ATM kinase, RAGE translocates to the nucleus and participates in DNA double-strand break repair, thereby helping prevent cellular senescence, tumorigenesis, and fibrosis (58). Furthermore, RAGE on endothelial cell surfaces can act as a ligand, mediating leukocyte adhesion and recruitment (59).

## 3 The effects of AGEs-RAGE axis in diabetic wounds

### 3.1 Peripheral neuropathy

Peripheral neuropathy, including the impairment of sensory and autonomic nerves, is a common complication among diabetic patients (60).

Sensory nerves extend into the epidermal layer, where they detect and transmit external stimuli to the central nervous system to initiate defensive responses. Beyond their sensory role, these nerves regulate skin growth and maintain homeostasis by secreting neurotrophic factors and neuropeptides (61). However, AGEs–RAGE activation induces inflammation and oxidative stress by the NF-κB-p65 pathway, which inhibits nerve growth and disrupts their secretory capacity (62, 63). Furthermore, AGEs–RAGE signaling promotes the activation of pro-inflammatory M1 macrophages, leading to inflammatory infiltration that contributes to neuronal insulin resistance and atrophy (64). Such damage elevates mechanical and thermal pain thresholds, reducing the body’s ability to respond effectively to external stimuli. Additionally, the loss of neurotrophic support adversely affects the renewal and functionality of keratinocytes, fibroblasts, and immune cells, thereby compromising skin repair (61, 65).

**Figure 3:**
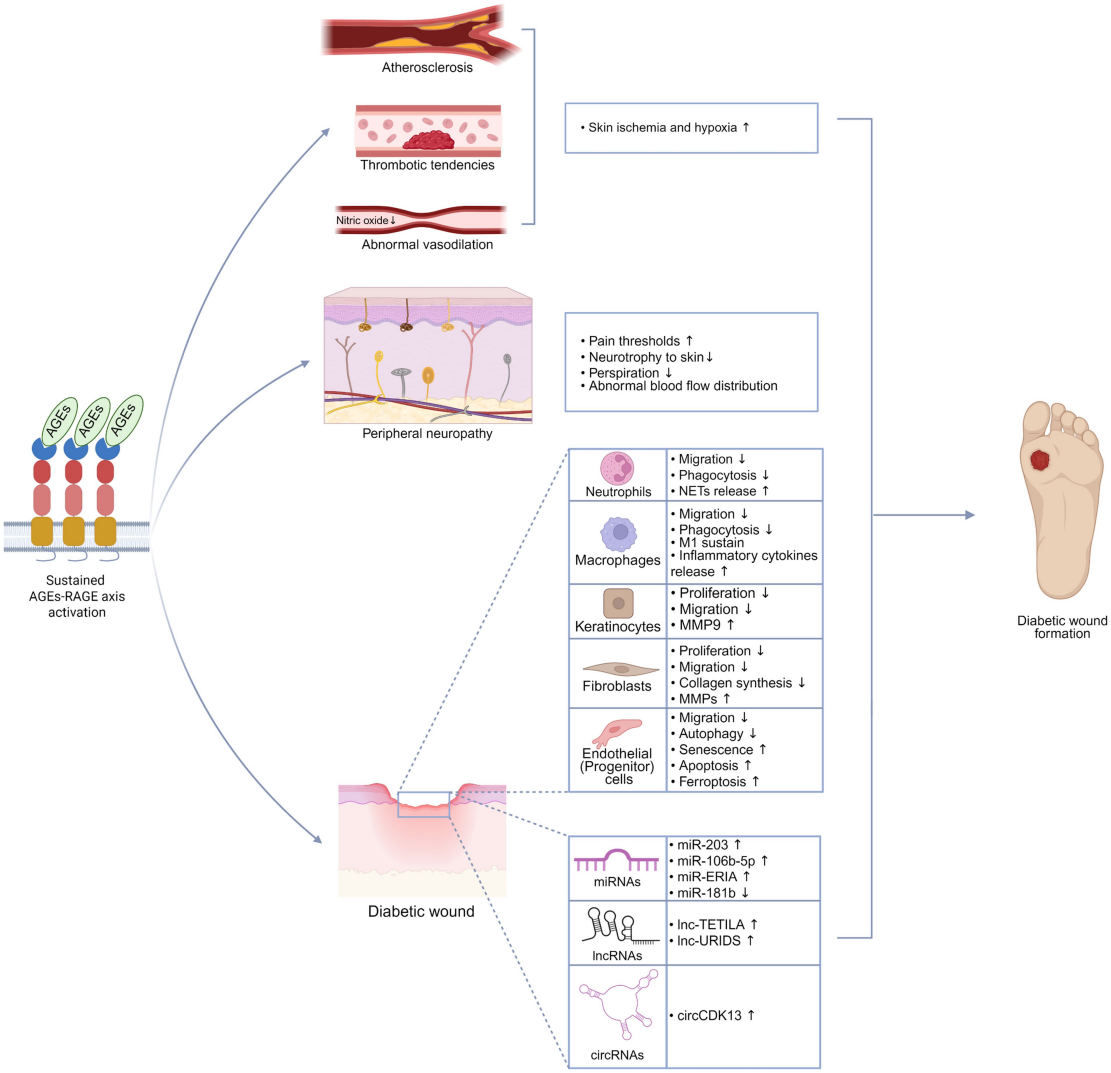
AGEs-RAGE axis promotes the formation of diabetic wounds. Sustained activation of the AGEs-RAGE axis drives a range of pathological alterations that accelerate the formation of diabetic wounds, including atherosclerosis, thrombotic tendencies, impaired vasodilation, peripheral neuropathy, dysfunction of repair cells, and abnormal expression of ncRNAs. This figure was created in https://BioRender.com.

Long-term activation of RAGE impairs the autonomic nervous system as well (66). Autonomic nerves in the skin primarily regulate vascular smooth muscles and sweat glands. Vasomotor dysfunction caused by autonomic neuropathy disrupts normal blood flow distribution, impairing skin perfusion. Atrophy of the autonomic nerves innervating the sweat glands reduces perspiration, causing skin dryness and increasing susceptibility to wound formation (67) (Figure 3).

### 3.2 Vascular lesions

Local skin ischemia and hypoxia, primarily resulting from vascular abnormalities such as atherosclerosis, endothelial dysfunction, and thrombotic tendencies, play a central role in the impaired healing of diabetic wounds. Activation of the RAGE pathway promotes the development of these pathological changes (68) (Figure 3).

Atherosclerosis is characterized by lipid deposition, fibrous proliferation, and calcification within the arterial intima, eventually leading to plaque formation and vascular narrowing. Atherosclerosis is prevalent in the lower extremities of diabetic patients and primarily affects the mid-sized arteries, such as the posterior and anterior tibial arteries (69). Therefore, diabetic wounds frequently manifest in the distal lower extremities, especially in the feet. The initial steps of atherosclerosis involve the accumulation of low-density lipoproteins (LDLs) beneath the arterial intima, where they are oxidized into oxidized LDLs (ox-LDLs) (70). AGEs promote LDL transcytosis across endothelial cells via the RAGE/NF-κB/Caveolin-1 axis, facilitating LDL deposition within the intima (71). Moreover, the oxidative stress induced by the AGEs-RAGE axis accelerates the oxidation of LDLs. Subsequently, macrophages migrate into the intima, engulf ox-LDLs, and transform into foam cells. AGEs–RAGE signaling upregulate the expression of cluster of differentiation 36 and Cyclin-dependent Kinase 5, promoting macrophage migration and enhancing the capacity to engulf ox-LDLs (72, 73). Furthermore, the AGEs-RAGE axis induces the expression of adhesion proteins such as intercellular adhesion molecule 1, vascular cell adhesion molecule 1, and E-selectin in endothelial cells, promoting the sustained retention of foam cells within the intima (74). Foam cells release substantial amounts of inflammatory factors that stimulate the migration, proliferation, and extracellular matrix (ECM) synthesis of vascular smooth muscle cells (VSMCs), leading to arterial intima thickening. Eventually, the plaque’s core consists of lipids, macrophages, and VSMCs (75). Progressive plaque enlargement reduces distal blood perfusion, directly contributing to wound formation and impairing wound healing.

Endothelial dysfunction, marked by impaired nitric oxide (NO)-mediated vasodilation, significantly influences blood supply to distal small vessels and exacerbates the narrowing of atherosclerotic vessels (76). NO production depends on endothelial nitric oxide synthase (eNOS). ROS induced through the AGEs-RAGE axis reduces the nuclear translocation of transcription factor EB, suppressing autophagic flux. This disruption contributes to eNOS uncoupling, reducing NO bioavailability and causing endothelial dysfunction (77, 78).

Thrombotic tendencies arise from increased platelet counts and hyperactivity, which can worsen atherosclerosis and occlude small blood vessels. RAGE expression is elevated in the platelets of diabetic and aging patients. The AGEs-RAGE axis enhances the activity and aggregation capacity of platelets, promoting a pre-thrombotic state (79, 80). Furthermore, the AGEs-RAGE axis upregulates endothelin-1 expression, which mediates vascular endothelial dysfunction and promotes thrombosis (81).

### 3.3 Dysfunction of cells for wound repair

#### 3.3.1 Neutrophils

Neutrophils are the first immune cells to arrive at wounds, primarily tasked with killing microbes. Overactivation of RAGE by AGEs disrupts the neutrophil extravasation and compromises their pathogen-killing abilities (82). Increased oxidative stress is one of the major factors leading to diabetic wounds. RAGEs mediate the generation of ROS in neutrophils via activation of NADPH oxidase (83). Neutrophils can release neutrophil extracellular traps (NETs) to capture bacteria and restrict the progression of infection. However, NETs contain various proteases, and their increase enhances the digestion of wound ECM, thereby promoting the formation of unhealing wounds (84) (Figure 3). The AGEs-RAGE axis enhances MEK/p38-MAPK/TGF-beta activated kinase 1 signaling, inducing citrullination of histone H3 at its R2 residue, thereby promoting the formation of NETs (85).

#### 3.3.2 Macrophages

Macrophages are the orchestrators of wound inflammation (86). In later stages of normal wound repair, macrophages transition from a pro-inflammatory M1 phenotype to a pro-reparative M2 phenotype, a shift critical for wound healing. RAGE activation, however, induces macrophages to retain a pro-inflammatory M1 phenotype by NF-κB signaling and ROS (87–89). Pyroptosis is a form of programmed cell death that leads to the release of intracellular pro-inflammatory substances. The AGEs-RAGE axis may exacerbate wound inflammation and impair wound healing by inducing the Caspase-11-dependent pyroptosis-related protein (90). Macrophages are also responsible for eliminating pathogens and cellular debris. Similar to neutrophils, their migratory and phagocytic functions are impaired by the AGEs-RAGE axis (Figure 3).

#### 3.3.3 Keratinocytes

Wound closure depends on re-epithelialization driven by keratinocyte migration and proliferation. The AGEs-RAGE axis inhibits keratinocyte proliferation, migration, epidermal lipid synthesis, and the expression of epidermal antimicrobial peptides by promoting inflammation and oxidative stress, thereby impairing epidermal regeneration and barrier function (91). Keratinocytes are a major source of MMP9. MMP9 is regarded as a promising therapeutic target, given its significant expression in diabetic wounds compared to other MMPs (92). MMP9 not only hinders wound healing through ECM degradation but also induces keratinocyte apoptosis *via* the FasL/Fas pathway (93). The AGEs-RAGE axis enhances MMP9 expression by promoting the demethylation of its gene promoter in keratinocytes (94). Furthermore, RAGE activation in keratinocytes leads to the abnormal secretion of Notch ligands. Overactivated Notch1 signaling pathway, in turn, induces NF-κB to promote MMP9 expression (95) (Figure 3).

#### 3.3.4 Fibroblasts

Dermal is the primary structural component of skin, and its repair largely relies on the proliferation, differentiation, and ECM remodeling by fibroblasts. Accumulated AGEs form stable cross-links with the main component of the ECM, collagen fibers, disrupting the normal collagen structure and leading to increased nano-stiffness and reduced hydration in the ECM (96–98). These cross-linked AGEs persistently interact with RAGE, compromising various fibroblast functions, including proliferation, migration, and collagen synthesis, while increasing secretion of MMPs (99). Moreover, a stiff ECM exacerbates chronic inflammation and accelerates cellular senescence (100). Senescent fibroblasts exhibit reduced contractility and impaired collagen secretion, adopting a senescence-associated secretory phenotype that releases inflammatory cytokines and MMPs (101) (Figure 3). These alterations further degrade the ECM and prolong inflammation.

#### 3.3.5 Endothelial cells

Angiogenesis driven by endothelial cells is crucial to wound healing. The RAGE activated by high levels of AGEs inhibits endothelial cell migration and promotes various forms of cellular dysfunction, including autophagy abnormalities, senescence, apoptosis, and ferroptosis (102, 103). Vascular endothelial growth factor (VEGF), a key angiogenic stimulant that promotes endothelial cell proliferation and migration, is downregulated in AGE-treated wounds *via* the RAGE pathway (104). Bone marrow-derived endothelial progenitor cells (EPCs), which serve as reservoirs for vascular repair, are also compromised by RAGE activation. The AGEs-RAGE axis disrupts autophagy and induces apoptosis in EPCs, thereby diminishing their ability to support angiogenesis and vascular repair (105, 106) (Figure 3).

### 3.4 Abnormal expression of ncRNAs

Non-coding RNAs (ncRNAs), including microRNAs (miRNAs, miR), long non-coding RNAs (lncRNA, lnc), and circular RNAs (circRNAs, circ), serve as critical regulators of gene expression through epigenetic, transcriptional, and post-transcriptional mechanisms (107). The AGE-RAGE axis induces aberrant expression of specific ncRNAs, leading to impaired cellular homeostasis and tissue repair processes. Examples include miR-203 and lnc-TETILA in keratinocytes (108, 109), lnc-URIDS and circCDK13 in fibroblasts (110), as well as miR-181b and miR-106b-5p in endothelial cells (111, 112) (Figure 3). Extracellular vehicles (EVs) function as crucial mediators of intercellular communication by transporting diverse cargo, including nucleic acids, proteins, and lipids (113). The AGEs-RAGE axis promotes specific ncRNAs expression that becomes packaged into EVs, subsequently influencing recipient cell behavior. Recent studies elucidated that AGEs promote macrophage polarization toward the pro-inflammatory M1 phenotype. These activated macrophages secrete EVs enriched with miR-ERIA, which targets helicase with zinc finger 2 in vascular endothelial cells, ultimately impairing endothelial migration and tube formation capacity (114). Further investigation of ncRNAs modulated by the AGEs-RAGE axis may provide potential therapeutic targets for enhancing diabetic wound healing.

## 4 Strategies targeting the AGEs-RAGE axis for diabetic wound repair

RAGE-induced chronic inflammation plays a critical pathogenic role in the development of diabetic wounds, positioning the AGEs-RAGE axis as a promising therapeutic target. Several strategies have been developed to directly inhibit this axis, including specific small molecule inhibitors, gene therapy targeting RAGE, monoclonal antibodies, AGE scavenger, and sRAGE. Here, we focus on the AGEs-RAGE axis-targeted interventions that have shown efficacy in diabetic wound healing (Table 1).

**Table 1:** Interventions for the AGEs-RAGE axis and their preclinical/clinical efficacy in diabetic wounds.

Strategies	Approaches	Route of administration	Preclinical/clinical efficacy	References
Small-molecule inhibitors of RAGE	FPS-ZM1	Topical application	Promote re-epithelialization, collagen deposition, and neovascularization, and alleviate inflammation in diabetic wounds of rats	Sun et al. (115)
	TPP488	Oral administration	Delay cognitive decline in AD patients	Burstein et al. (117)
	RAGE229	Topical application	Facilitate wound healing in type 2 diabetic mice	Manigrasso et al. (118)
RAGE antibodies	Monoclonal antibody	Intramuscular injection	Improve wound healing in diabetic pigs	Johnson et al. (120)
	RAGE vaccine	Subcutaneous injection	Induce sufficient generation of RAGE-IgG antibodies and improve the renal function in diabetic mice	Azegami et al. (121)
Gene therapy for RAGE	RAGE-siRNA	Local delivery by bioengineering carriers	Alleviate acute myocardial injury; improve multiple diabetic complications	Yang et al. (124), Park et al. (125), Cai et al. (128)
	miR-5591-5p	Lentiviral transfection	Enhance the ability of ADSCs to repair the wounds of diabetic mice	Li et al. (130)
	miR-23a-5p	Tracheal instillation of miR-23a-5p agomir	Improve lung function in COPD mice	Chang et al. (134)
	miR-1915	Not applicable	Not applicable	Xu et al. (133)
	miR-328-5p	Not applicable	Not applicable	Luo et al. (131)
	miR-185-5p	Transfection by liposome	Inhibit the invasion of breast cancer cells in mouse model	Yin et al. (132)
Specific AGEs scavengers	Aminoguanidine	Topical application	Promote the healing of tissue-engineered diabetic wounds	Lemarchand et al. (139)
	Alagebrium (ALT-711)	Oral administration/topical application	Promote foot ulcer healing in the diabetic rat model	Harb et al. (140)
	Magnetically driven nanorobots for scavenging AGEs	Not applicable	Not applicable	Yuan et al. (141)
	Chiral gel dressing HA-LM2-RMR	Topical application	Facilitate angiogenesis and re-epithelialization in the diabetic wound of mice	Xing et al. (142)
	Zeolitic imidazolate framework-8 encapsulated cerium dioxide and adsorbed glucose oxidase nanozyme particles	Topical application	Increase collagen deposition and accelerate wound closure	Zhang et al. (143)
Non-specific RAGE ligand scavengers	sRAGE	Topical application	Enhance multiple growth factors expression, promote collagen deposition, and angiogenesis in diabetic mice	Goova et al. (144)
Anti-inflammatory and antioxidant substances for inhibiting the RAGE pathway	Resveratrol	Topical application	Promote wound healing in diabetic mice and attenuate the expression of pro-inflammatory cytokines	Youjun et al. (147)
	Gallic acid	Topical application	Accelerate wound healing in diabetic mice	Chen et al. (148)
	Bitter melon extract	Not applicable	Not applicable	Aljohi et al. (150)
	Gentiopicroside	Not applicable	Not applicable	Chen et al. (151)
	Rosiglitazone	Topical application	Promote wound healing in diabetic rats	Yang et al. (157)
	Arabic gum	Topical application	Promotes diabetic wound healing in rats	Chai et al. (167)
Healthy lifestyle and dietary habits	Low-AGE diet, regular exercise, smoking cessation, and alcohol moderation	Not applicable	Not applicable	Portero-Otin et al. (163), Luo et al. (164), Lu et al. (165), Fotheringham et al. (166)

### 4.1 Small molecule RAGE inhibitors

Several small-molecule inhibitors targeting the extracellular or intracellular domains of RAGE have been developed.

FPS-ZM1 is a non-toxic tertiary amide compound that binds to the V-domain of RAGE to block its interaction with ligands. Recently, Sun et al. (115) designed a cobalt (Co)-based metal–organic framework loaded with FPS-ZM1 nanoparticles. This complex material, through localized release of FPS-ZM1 and Co-ions, promoted re-epithelialization, collagen deposition, and neovascularization, along with alleviating inflammation in diabetic wounds in rats. TTP488/Azeliragon is another inhibitor binding to the extracellular domain of RAGE, effectively preventing the interaction of RAGE with various ligands, including AGEs, HMGB1, S100, and Aβ (116). TTP488 is currently the only RAGE inhibitor undergoing clinical trials. In a Phase IIb clinical trial for mild-to-moderate Alzheimer’s disease patients, an 18-month treatment with TTP488 significantly delays cognitive decline compared to placebo (117). Further exploration of its application for wound treatment holds potential appeal.

RAGE229 is a novel small molecular inhibitor to block the interaction between the intracellular domain of RAGE and DIAPH1. Topical application of RAGE229 has been demonstrated to significantly accelerate wound healing in type 2 diabetic mice (118).

### 4.2 RAGE antibodies

Monoclonal antibodies targeting RAGE block signal transduction by directly binding to the extracellular domain of the receptor. Johnson et al. (119) developed a monoclonal IgG antibody against RAGE, named CR-3. In porcine models of diabetic wounds, the intramuscular injection of CR-3 at a dosage of 1 mg/kg every 10 days enhanced wound healing than non-immune IgG treatment (120). Interestingly, CR-3-loaded decellularized porcine skin patches showed no difference compared to the normal patches, likely due to poor retention or inadequate local concentration of the macromolecular antibody within the patch material (120). However, it is important to emphasize that the high cost of monoclonal antibody production poses a challenge to their clinical application in diabetic wounds. In contrast, vaccines may represent a more cost-effective approach. A vaccine designed to stimulate the production of RAGE antibodies has been developed. These antibodies selectively bind to the AGE-specific site on RAGE (amino acids 38–44), blocking AGE-driven signaling without affecting other RAGE ligands, resulting in partial inhibition of the pathway (121). In diabetic mice, three rounds of immunization with this vaccine successfully induced sufficient titers of specific IgG antibodies for at least 38 weeks. Treated mice exhibited improvements in renal function and histopathology (121), but the effects on diabetic wound healing have yet to be investigated. In addition, the long-term safety and efficacy profile of this vaccination strategy requires further evaluation.

### 4.3 Gene therapy for RAGE

Reducing the expression of RAGE through gene therapy is a promising approach (122). Small interfering RNA (siRNA) is a short double-stranded RNA that integrates into the RNA-induced silencing complex, targeting complementary messenger RNA (mRNA) for degradation to suppress gene expression (123). The knockdown of RAGE using siRNA has shown significant efficacy in animal models of diabetic wounds and pathological scars (57, 88). To enhance delivery efficiency and mitigate the concern of gene integration associated with viral vector delivery, several non-viral vectors for delivering RAGE siRNA have been developed, including deoxycholic acid-modified polyethyleneimine, bio-reducible polyethyleneimine, and EVs (124–126). Tetrahedral framework nucleic acid (tFNA) is an innovative carrier composed of four single-stranded DNA molecules, exhibiting potent cell membrane penetrability, high biosafety, and nucleases resistance (127). Cai et al. (128) recently developed a tFNA-based siRNA delivery system targeting RAGE, which effectively ameliorated various diabetic complications in animal models. miRNA is another type of small, single-stranded RNAs that regulate mRNA translation by binding to complementary sequences (129). miR-5591-5p has been identified to enhance the wound healing capacity of adipose tissue-derived stem cells in diabetic conditions by targeting RAGE mRNA (130). Moreover, miR-23a-5p, miR-1915, miR-328-5p, and miR-185-5p can also regulate RAGE mRNA (131–134). Further investigation into alternative gene silencing techniques, such as short hairpin RNA (shRNA), antisense oligonucleotides, and CRISPR/Cas9, in conjunction with other innovative delivery systems, including liposomes, hydrogels, microneedles, and micelles, would be advantageous in broadening therapeutic approaches for RAGE gene therapy (135).

Targeting the RAGE exhibits substantial potential in diabetic wound healing. However, given RAGE’s critical roles in physiological processes, complete blockade via vaccines or gene manipulation may impair its homeostatic functions. Increased abscess formation and delayed wound healing are observed in the RAGE knockout mice (136). Therefore, more experiments are needed to discover the potential adverse effects of long-term blockade of the RAGE pathway. Localized delivery and transient inhibition may contribute to mitigating such risks (137).

### 4.4 Targeting AGEs

In addition to directly targeting RAGE, another effective strategy for limiting RAGE pathway activation is enhancing the clearance of its ligands.

Several specific approaches targeting AGEs clearance have been identified to promote the healing of diabetic wounds. Aminoguanidine is a classic AGE scavenger that reduces the production of AGEs by neutralizing carbonyl compounds (138). It has been shown to reverse AGE-induced non-healing of wounds in tissue-engineered models (139). Alagebrium (ALT-711) is an AGE crosslink breaker. Systemic or local treatment with Alagebrium improves wound healing in diabetic rat models (140). Some novel bioactive materials are designed with binding sites for AGEs to facilitate their clearance (141–143). For example, a gel formed from L-phenylalanine and cationic hexapeptide co-assembled helical nanofibers cross-linked with hyaluronic acid possesses abundant chiral and cationic sites, effectively reducing AGEs through stereoselective interactions (142).

sRAGE non-specifically binds to RAGE ligands, obstructing their interaction with RAGE and consequently inhibiting RAGE signaling. Localized application of sRAGE significantly reduces inflammation in diabetic wounds while also enhancing the expression of multiple growth factors, promoting collagen deposition, and accelerating angiogenesis (144). However, sRAGE is prone to degradation in the protease-rich environment of wounds. Kang et al. (145) developed a recombinant fusion protein containing the V-domain of RAGE (vRAGE) linked to elastin-like polypeptides (ELPs). The vRAGE domain retains the ability to bind RAGE ligands, while the ELP domain enables temperature-dependent self-assembly into coacervates at around 30–31 °C, thereby shielding vRAGE from degradation and facilitating its sustained release. This fusion protein remains stable in elastase *in vitro* for up to 7 days and has demonstrated efficacy in promoting wound healing in diabetic mice.

### 4.5 Other approaches for the AGEs-RAGE axis inhibition

#### 4.5.1 Pharmacological regulation

Another method for inhibiting the AGEs-RAGE axis involves natural or synthetic anti-inflammatory and antioxidant drugs along with diabetic management agents.

Polyphenolic compounds can scavenge ROS and inhibit the nuclear translocation of NF-κB to reduce inflammation (146). These effects significantly disrupt RAGE signaling and suppress RAGE expression. Polyphenolic compounds, including resveratrol and gallic acid, have been found to promote diabetic wound healing by inhibiting the AGEs-RAGE axis (147, 148). Steroidal saponins also suppress the RAGE pathway through their anti-inflammatory and antioxidant properties (149). Bitter melon extract, rich in steroidal saponins, effectively counteracts the inhibition of angiogenesis caused by the AGEs-RAGE axis in diabetic wounds (150). Gentiopicroside, a cleaved-ring enol ether terpene glycoside, was recently discovered to protect against glycation-induced damage in skin fibroblasts by inhibiting the AGEs-RAGE axis (151). Furthermore, anti-inflammatory and antioxidant substances such as flavonoids, natural polysaccharides, ascorbic acid, and tocopherol are also associated with the inhibition of the RAGE pathway (152–155). However, additional research is required to validate their therapeutic effects on diabetic wounds.

The classic anti-diabetic agent rosiglitazone inhibits the harmful effects of the AGEs-RAGE axis by activating the peroxisome proliferator-activated receptor gamma pathway (156). Topical application of a hydrogel dressing loaded with nanoparticles containing rosiglitazone and S-nitrosoglutathione markedly promotes wound healing in diabetic rats (157). Moreover, metformin, dipeptidyl peptidase-4 inhibitors, and glucagon-like peptide-1 analogs, primarily used for type 2 diabetes management, may also facilitate diabetic wound closure by suppressing RAGE-mediated inflammation (158–162).

#### 4.5.2 Lifestyle interventions

Lifestyle and dietary habits significantly influence the levels of AGEs. AGEs are particularly abundant in red meat, dairy products, processed foods, high-sugar diets, and foods prepared using high-temperature cooking methods such as frying, grilling, and roasting. In contrast, fruits, vegetables, whole grains, and foods prepared using moist-heat cooking techniques (poaching, steaming, and stewing) contain lower AGEs (163). Beyond diet, regular physical activity, smoking cessation, and moderate alcohol consumption constitute important approaches for reducing AGE accumulation *in vivo* (164–166). These lifestyle improvements may serve as effective adjuncts in the prevention and treatment of diabetic wounds.

## 5 Conclusion and future perspective

The AGEs-RAGE axis serves as a crucial mediator in diabetic wound pathogenesis by driving chronic inflammation, oxidative stress, and cellular dysfunction through sustained activation of multiple inflammatory and oxidative stress pathways. Current intervention strategies, including RAGE inhibitors, gene silencing, AGE scavengers, various anti-inflammatory and antioxidant drugs, and several anti-diabetic agents, demonstrate preclinical efficacy in restoring angiogenesis, resolving inflammation, and promoting tissue repair. Dietary and lifestyle interventions further complement these approaches by reducing systemic AGE accumulation. However, the transition from preclinical success to clinical application remains a critical challenge, necessitating rigorous trials to validate safety and efficacy in diverse diabetic populations.

Future research should prioritize cost-effective, targeted therapies, such as innovative delivery systems and multimodal combinatorial regimens. Notably, the rapid advancements in artificial intelligence in recent years are expected to accelerate the discovery and development of more effective AGEs-RAGE axis-targeted drugs. Interdisciplinary collaboration and public health initiatives are essential to bridge mechanistic insights with transformative clinical outcomes, ultimately improving the quality of life for patients with diabetic wounds.
